# Magnitude of Neonatal Near Miss and Associated Factors in Hospitals of South West Shoa Zone, Central Ethiopia: A Cross‐Sectional Study

**DOI:** 10.1002/hsr2.71504

**Published:** 2025-11-17

**Authors:** Shiferaw Benti, Tsegaye Benti Muse, Kisu Meskele Telila, Rahel Abdissa, Elias Teferi Bala

**Affiliations:** ^1^ Department of Public Health, Ambo University College of Health Sciences and Referral Hospital Ambo Oromia Ethiopia; ^2^ Department of Pediatric and Child Health, Ambo University College of Health Sciences and Referral Hospital Ambo Oromia Ethiopia

**Keywords:** magnitude, near‐miss, neonatal, South West Shoa

## Abstract

**Background:**

Every year, almost 30 million neonates worldwide require hospitalization as a result of life‐threatening situations. Neonatal mortality rate occurrences can have long‐term negative impacts on a child's and their family's health, well‐being, and lifespan. Ethiopia has made efforts to increase infant survival, although the rate of neonatal death remains high.

The purpose of this study was to evaluate the frequency of neonatal near‐misses and related variables in South West Shoa Zone hospitals in Central Ethiopia in 2022.

**Methods:**

From September 9 to November 8, 2022, an institution‐based cross‐sectional study was carried out in the Southwest Shoa Zone. Three hundred fifty‐six mothers and their corresponding newborns were carefully chosen, and the interviewer delivered a structured questionnaire along with a review of the mothers’ and newborns’ medical records to gather the data. Bivariate and multivariable logistic regression analyses were employed to determine the factors linked to neonatal near misses.

**Findings:**

The neonatal near‐miss had a magnitude of 29.2% (95% CI = 24.47%, 33.96%). Pregnancy‐induced hypertension (AOR: 3.07; 1.15, 8.14), non‐vertex presentation (AOR = 5.27; 1.83, 15.16), antepartum hemorrhage (AOR = 5.28; 1.72, 16.18), premature rupture of membrane (AOR = 4.03; 1.88, 8.64), inadequate prenatal care visit (AOR = 2.66; 1.49, 4.76), and cesarean section delivery (AOR = 2.96; 1.28, 3.87) were found to be significant predictors of neonatal near miss.

**Conclusion:**

According to this study, almost three out of ten newborns born in hospitals have a neonatal near‐miss that is influenced by multiple factors. To lower neonatal near misses, it is crucial to improve prenatal care visits and to identify and treat obstetric issues early.

## 1 Introduction

The term “neonatal near miss” (NNM) refers to a situation in which newborns survive due to either chance or the standard of care they get [1]. The neonatal phase is crucial for the neonates’ survival. Nearly 2 million babies die within the first 7 days of their lives, and more than one in three die on the day of birth [2].

Every year, almost 30 million babies worldwide require hospitalization as a result of life‐threatening situations. Newborns who do not receive the care they need may die, acquire acute or chronic illnesses, or experience delayed development due to low birth weight (LBW), preterm, congenital abnormalities, or sickness [3].

The burden of neonatal deaths is not evenly distributed among countries and regions. Nearly 80% of neonatal deaths in 2019 happened in two areas: 37% happened in Central and Southern Asia, and 42% happened in sub‐Saharan Africa. Due to increased birth rates and a minor decrease in the neonatal mortality rate (39 percent between 1990 and 2019), Sub‐Saharan Africa is the only SDG zone where there was no decrease in the number of neonatal deaths between 1990 and 2019. Between 1990 and 2019, the total number of neonatal fatalities in sub‐Saharan Africa was constant at almost 1 million, with 21 of the 48 countries experiencing no drop in neonatal mortality [4].

Few research have been conducted to demonstrate the burden of NNM in Africa. The prevalence of NNM was 36.7% in Ghana and 69.5% in Uganda, according to studies conducted in both countries [5, 6]. Similar research was done in Ethiopia to demonstrate the prevalence of NNM in various regions, including Injibara (23.3%), Debretabor (32.9%), Jimma (26.7%), and Hawassa City (33.4%).

Neonatal morbidity and mortality are still high despite Ethiopia's implementation of important neonatal and child survival interventions, including skilled birth attendance, postpartum care, immunization, vitamin A supplementation, solving the unmet need for family planning, improving care quality, expanding community‐based newborn care and quality facility new‐born care, increasing resources for health financing, including charge‐free services for maternal and neonatal care, and putting more emphasis on research [7, 8].

It is crucial for stakeholders and policymakers to evaluate the severity and contributing causes of neonatal near misses (NNM). This offers data that aids in the planning and execution of interventions to enhance perinatal outcomes by hospital administrators and zonal health departments. Furthermore, this study provides evidence to help healthcare providers better understand the scope and contributing aspects of the issue and to improve the health of newborns. To implement suitable actions at the community and health system levels, it also aids in identifying the contributing causes to newborn morbidity.

## 2 Methods

### 2.1 Study Setting

An institution‐based cross‐sectional study was conducted among 356 neonates with their mothers in hospitals of the South‐West Shoa zone, Oromia Region. The capital city of the zone is Waliso, which is located 114 km away from Addis Ababa on the way to Jimma. Based on the projection of the 2007 census conducted by the Central Statistical Agency of Ethiopia, the zone had a total of 1,101,129 people, of whom 556,194 were men and 544,935 were women. Southwest Showa Zone comprises five government hospitals named Tullu Bollo General Hospital, Weliso General Hospital, Ameya Primary Hospital, Bantu Primary Hospital, and Leman Primary Hospital, and one nongovernment hospital, Saint Luke's Catholic Hospital. It also had 54 health centers, 264 health posts, 72 private clinics, and 28 drug stores.

The study was conducted from September 9 to November 8, 2022.

### 2.2 Study Design

A community‐based cross‐sectional study was conducted.

### 2.3 Source and Study Population

All neonates who were born in hospitals of Southwest Shoa Zone were the source population, and the study population was all randomly selected neonates in the hospitals.

#### 2.3.1 Inclusion

All live births delivered in the selected hospitals and within 28 days of the neonatal period during the data collection period in hospitals were included.

### 2.4 Exclusion

Multiple pregnancies, neonates whose mothers died, and neonates who were referred from other health facilities other than the selected hospitals were excluded.

### 2.5 Sample Size Determination and Sampling Procedure

A single population proportion formula, *n* = (Zα/2)2*pq/d2, was used to calculate the sample size. The following assumptions were made: a 95% confidence level, 5% margins of error, a 10% nonresponse rate, and an NNM magnitude of 26.7%, which was taken from a study done in Jimma zone [9]. This resulted in a total sample size of 350. However, the twofold population proportion approach was used to determine the sample size for the following factors: pregnancy‐induced hypertension [10], antepartum hemorrhage [11], advanced maternal age > 35 years [12], and NNM. A comparatively higher sample size of 359 was obtained by applying the following assumptions: 80% power, a 95% confidence level, and a 1:1 exposed to unexposed ratio. Based on the availability of neonatal intensive care unit (NICU) services, five hospitals out of the six hospitals in the Southwest Shoa Zone were chosen using a systematic random sampling technique to select neonates with their respective mothers using the order of delivery registration logbook. Weliso General Hospital was thus disqualified from the study since it lacked an acute care unit. Based on the projected number of average live births over a 2‐month period—which was calculated from the annual report of the year before the study period—the sample size was distributed proportionately across the five hospitals. As a result, Saint Luke's Catholic Hospital, Tullu Bollo Hospital, Ameya Hospital, Bantu Hospital, and Leman Hospital had, respectively, 668, 334, 142, 78, and 132 live births on average over two consecutive months. By dividing the sample size (359) by the projected number of 2‐month live births (1354), the sampling interval (k) was determined to be roughly 4. Out of the first four newborns, the first one was chosen by lottery. Then, until the necessary sample size was reached, every fourth neonate was selected.

### 2.6 Data Collection

A pre‐tested, interviewer‐administered questionnaire with four sections—sociodemographic traits, obstetric history and problems, prenatal medical history, and neonatal factors—was used to gather data. Five BSc midwives with expertise in obstetric care and fluency in the local language, Afaan Oromoo, gathered the data. Two senior BSc midwives oversaw every step of the data collection process. Supervisors and data collectors received 2 days of training on data gathering procedures. Five percent of the entire sample size was pre‐tested at Inchini Primary Hospital, and any necessary adjustments were performed. At the conclusion of each data‐collecting day, the investigator and supervisors promptly verified that all of the data had been obtained. Training was provided to inform data collectors about bias to reduce interviewer‐administered prejudice, and daily debriefing sessions were held to identify any bias and promptly resolve it.

#### 2.6.1 Measures

Neonatal near miss: NNM was taken into consideration when the baby survived despite encountering at least one of the following suggested criteria (either pragmatic or management criteria). Using practical standards: 5th‐minute APGAR score < 7, gestational age < 33 weeks, birth weight < 1750 g, or The following are management criteria: nasal continuous positive airway pressure (NCPAP), parenteral therapeutic antibiotics for up to 7 days and before twenty‐8 days of life, any intubation during the first twenty‐8 days of life, and phototherapy within the first twenty‐4 h of life, cardiopulmonary resuscitation, any surgery performed on a newborn in the early stages of life, the use of vasoactive medications, anticonvulsants, surfactants, blood products, and steroids for refractory hypoglycemia, and parenteral nutrition [2]. A newborn's tone, color, respiration, pulse rate, and responsiveness are all factors that go into an APGAR score, which ranges from 0 to 10.

### 2.7 Data Analysis

After being coded and imported into Epi Info version 7.2.2.6, the gathered data were transferred to SPSS version 26 for analysis. First, frequencies were run to perform descriptive analysis. The relationship between the independent variables and the outcome variable was examined using both bivariate and multivariable logistic regression models. Bivariate variables that had a *p*‐value of 0.25 or less were moved to multivariable analysis. This makes it possible to include a wider variety of significant factors that might not be significant at the strict threshold level, like 0.05. It lessens the possibility of leaving out important variables that might be missed because of chance fluctuations. The variance inflation factor (VIF), which measures multi‐collinearity among independent variables, was less than 10, indicating that multi‐collinearity did not exist. In multivariable analysis, the confounders were controlled using a multivariable logistic regression model with the entry technique. To assess the model's fitness, a Hosmer‐Lemeshow goodness‐of‐fit test was performed; the results showed that the model fit well, with an insignificant *p*‐value of 0.315. The independent variables linked to the dependent variable were determined using the adjusted odds ratio (AOR), which has a 95% confidence interval. A p‐value of less than 0.05 was used to indicate statistical significance for all statistical tests.

#### 2.7.1 Ethical Considerations

The College of Health Sciences and Referral Hospital's Ethical Review Board at Ambo University granted ethical approval. Following the submission of the ethical clearance, a letter of support was acquired from the Oromia Regional Health Bureau. After that, the Zonal Health Office and each of the chosen hospitals received the letter of support. Each respondent was given an explanation of the study's purpose before providing their individual informed written consent. The identity of the interviewee was not recorded, and all answers were kept private. In this study, participation was entirely voluntary. Participants were made aware that they might leave the study at any moment and that they were not obligated to respond to any questions they did not want to. To protect participant confidentiality, all names and identifiers were provided in the questionnaires.

## 3 Results

### 3.1 Socio‐Demographic Characteristics of the Respondents

The survey comprised 356 mothers and their babies out of 359, yielding a 99.2% response rate. Seventy‐two percent of the mothers were between the ages of 20 and 34, with a mean age of 25.75 (± 5.528). Most of them were housewives, with 259 (72.8%) and 351 (98.6%) being married. Furthermore, 110 moms (30.9%) and 185 mothers (52%) were from rural areas and did not have a formal education, respectively (Table 1).

**Table 1:** Socio‐demographic characteristics of mothers of neonates delivered in hospitals of South‐West Shoa zone, Oromia, Central Ethiopia, 2022 (*n* = 356).

Characteristics	Frequency (*n*)	Percent %
Age of mother		
< 20	44	12.4
20–34	275	77.2
≥ 35	37	10.4
Marital status		
Married	351	98.6
Single/Divorced/widowed	5	1.4
Place of residence		
Urban	171	48
Rural	185	52
Religion		
Orthodox	176	49.4
Muslim	47	13.2
Protestant	130	36.5
Others	3	0.84
Maternal educational status		
No formal education	110	30.9
Primary (1–8)	151	42.4
Secondary (9–12)	53	14.9
College and above	42	11.8
Partner educational status		
No formal education	54	15.2
Primary (1–8)	155	43.5
Secondary (9–12)	82	23
College and above	65	18.3
Maternal occupation		
House wife	259	72.8
Merchant	38	10.7
Government employee	37	10.4
Private employee	20	5.6
Daily laborer	2	0.6
Partner occupation		
Farmer	166	46.6
Merchant	73	20.5
Government employee	67	18.8
Private employee	42	11.8
Daily laborer	8	2.2
Average monthly income		
< 2000	112	31.5
2001–3500	99	27.8
3501–5000	102	28.7
> 5000	43	12.1

### 3.2 Maternal Obstetric and Medical Factors

Of the mothers, 186 (47.2%) were multiparous, and 41 (11.55%), 24 (6.7%), 15 (4.2%), and 11 (3.11%) had a history of either preterm labor, stillbirth, abortion, or neonatal mortality. Ninety‐four percent of the mothers of the newborns received at least one antenatal care (ANC) follow‐up visit, and 125 (35.1%) had less than four visits during their current pregnancy. Seventy‐one percent of the mothers gave birth naturally by vaginal delivery. As for chronic illness, during their present pregnancies, 12 (3.4%) and 18 (5.1%) of the women, respectively, had syphilis and anemia (Table 2).

**Table 2:** Maternal obstetric and medical factors among mothers of the neonates delivered in hospitals of South West Shoa zone, Oromia, Central Ethiopia, 2022 (*n* = 356).

Characteristics	Frequency	Percent %
I. Reproductive History		
Parity		
Primiparous	141	39.6
Multiparous	168	47.2
Grand multiparous	47	13.2
History of previous abortion		
Yes	41	11.5
No	305	88.5
Birth interval		
< 24 months	75	21.1
≥ 24 months	160	44.9
NA	121	34
ANC follow up		
Yes	336	94.4
No	20	5.6
Number of ANC visits		
1–3	125	35.1
≥ 4	211	59.3
II. Obstetric complications		
APH		
Yes	23	6.5
No	333	93.5
PROM		
Yes	51	14.3
No	305	85.7
Abnormal labor progress/Dystocia		
Yes	43	12.1
No	313	87.9
Type of abnormal labor		
Prolonged labor	26	7.5
Cephalo‐pelvic disproportion	14	3.9
Uterine rupture/dehiscence	3	0.8
Pregnancy‐induced hypertension		
Yes	30	8.4
No	326	91.6
Mode of delivery		
Spontaneous vaginal Delivery	253	71.1
Instrumental delivery	47	13.2
Caesarean Section	56	15.7
III. Medical conditions		
Anemia		
Yes	18	5.1
No	338	94.9
Syphilis		
Yes	12	3.4
No	344	96.6
HIV/AIDS		
Yes	3	0.84
No	353	99.16

Abbreviations: ANC, antenatal care; APH, antepartum hemorrhage; PROM, premature rapture of membrane.

### 3.3 Neonatal Factors

Among the newborns, 184 (51.7%) were male, and 330 (92.7%) had a vertex presentation at birth. Of the newborns, 238 (66.9%) were born between 37 and 41 weeks, 81 (22.8%) were born before 37 weeks, and the remaining 37 (10.4%) were delivered after 42 weeks. Out of all the newborns, 307 (86.2%) were born weighing normal, 33 (9.3%) weighing less than 2499 grams, and 16 (4.5%) weighing more than 4000 grams. Of the newborns, 94 (26.4%) were admitted to the NICU.

### 3.4 Magnitude of Neonatal Near Miss

The neonatal near‐miss magnitude in this study was 104 (29.2%) (95% CI: 24.47–33.96%). The most common pragmatic and treatment criteria were intravenous antibiotic use for up to 7 days in 69 (66.3%) of the neonates and a 5th minute APGAR score of less than 7 in 76 (73%) of the neonates. Of the criteria used to define NNM, this study did not identify the management requirements for surfactant use, parenteral nutrition, or surgical treatments; about 85.7% of the neonates met multiple criteria (Table 3).

**Table 3:** Distribution of NNM based on pragmatic or management criteria among neonates delivered in hospitals of Southwest Shoa Zone, Oromia, Central Ethiopia, 2022 (*n* = 104).

Neonatal near miss conditions	Frequency *n*	Percent %
Pragmatic criteria		
Gestational age < 33 weeks	11	10.6
Birth weight < 1750 gm	8	7.7
5th minute APGAR score < 7	76	73
Management criteria		
Use of parenteral antibiotics up to 7 days	69	66.3
Use of nasal CPAP	26	25
Use of intubation	3	2.9
Use of phototherapy in the first 24 hr	4	3.8
Cardiorespiratory resuscitation	43	41.3
Use of vasoactive drugs	5	4.8
Use of anticonvulsant	15	14.4
Use of blood products	2	1.9
Use of corticosteroid for treatment of refractory hypoglycemia	9	8.7
Feeding problems (NG‐tube feeding)	31	29.8
Congenital malformation	3	2.9

Abbreviations: APGAR, appearance, pulse, grimace, activity, respiration; CPAP, continuous positive airway pressure; NG, naso‐gastric*.*

The total percentage adds up to > 100% because some of the neonates had more than one from pragmatic or management criteria.

### 3.5 Factors Associated With Neonatal Near Miss

Maternal age, maternal education, residence, number of ANC visits, ante‐partum hemorrhage (APH), premature rupture of membrane (PROM), difficult/abnormal labor, mode of delivery, pregnancy‐induced hypertension, anemia during the current pregnancy, and fetal presentation at birth were all candidates for the multivariable logistic regression model after demonstrating an association at a *p*‐value of 0.25 in the bi‐variable logistic regression. The following covariates were found to be substantially linked with NNM in multivariable logistic regression analysis: ANC visit, APH, PROM, method of delivery, pregnancy‐induced hypertension, and fetal presentation at birth (*p*‐value < 0.05).

The odds of developing NNM were 2.66 times greater for mothers who had insufficient ANC visits during their current pregnancy than for mothers who had adequate ANC visits (AOR = 2.66; 95% CI:1.49, 4.76). Neonates born to mothers with APH were 5.285 times more likely to experience the NNM condition than neonates born to mothers without APH (AOR = 5.28; 95% CI: 1.72, 16.18). Neonates born to mothers with PIH had three times the chance of having NNM compared to those born to mothers without PIH (AOR = 3.07; 95% CI: 1.15, 8.14).

Furthermore, neonates whose mothers had a history of PROM were four times more likely to experience NNM than neonates whose mothers had no history of PROM (AOR = 4.03; 95% CI: 1.88, 8.64). The likelihood of NNM was 2.9 times higher for neonates delivered by cesarean section than for those delivered vaginally (AOR = 2.96; 95% CI: 1.28, 3.87). Additionally, this study demonstrated that neonates with non‐vertex presentation at delivery had a 5.27‐fold increased risk of experiencing a NNM episode in comparison to those with vertex presentation (AOR = 5.27; 95% CI: 1.83, 15.16) (Table 4).

**Table 4:** Bi‐variable and multivariable logistic regression analyses for factors associated with NNM among neonates delivered in hospitals of Southwest Shoa Zone, Oromia, Central Ethiopia, 2022 (*n* = 356).

Variables category	Neonatal near miss		COR (95%)	AOR (95% CI)	*p*‐value
	Yes (%)	No (%)			
Maternal age					
< 20	12 (27.3)	32 (72.7)	0.96 (0.04–1.96)	1.161 (0.48–2.78)	0.737
20–34	77 (28)	198 (72)	1	1	
≥ 35	15 (40.5)	22 (59.5)	1.75 (0.86–3.55)	0.89 (0.33–2.39)	0.831
Residence					
Urban	41 (24)	130 (76)	1	1	
Rural	63 (34)	122 (66)	1.63 (1.02–2.6)	1.7 (0.87–3.3)	0.116
Maternal educational level					
No formal education	42 (38.2)	68 (61.8)	1.83 (1.13–2.96)	1.29 (0.62–2.68)	0.489
Had formal education	62 (25.2)	184 (74.8)	1	1	
ANC visit *n *= 336					
1–3	57 (45.6)	68 (54.4)	3.69 (2.25–6.06)	2.66 (1.49–4.76)	0.001
≥ 4	39 (18.5)	172 (81.5)	1	1	
APH					
Yes	16 (69.5)	7 (30.5)	6.36 (2.53–15.98)	5.28 (1.72–16.18)	0.001
No	88 (26.4)	245 (73.6)	1	1	
PROM					
Yes	28 (54.9)	23 (45.1)	3.66 (1.99–6.74)	4.03 (1.18–8.64)	0.001
No	76 (24.9)	229 (75.1)	1	1	
Dystocia					
Yes	22 (51.2)	21 (48.88)	2.95 (1.54–5.64)	1.84 (0.73–4.6)	0.191
No	82 (26.2)	231 (73.8)	1	1	
Mode of delivery					
SVD	54 (21.3)	199 (78.7)	1	1	
Instrumental	14 (29.8)	33 (70.2)	1.56 (0.78–3.12)	1.27 (0.52–3.08)	0.59
C/S	36 (64.3)	20 (35.7)	6.63 (3.55–12.37)	2.96 (1.28–6.87)	0.011
PIH					
Yes	19 (63.3)	11 (36.7)	4.89 (2.23–10.71)	3.07 (1.15–8.34)	0.024
No	85 (26.1)	241 (73.9)	1	1	
Anemia					
Yes	8 (44.4)	10 (55.6)	2.01 (0.77–5.26)	2.26 (0.61–8.34)	0.218
No	96 (28.4)	242 (71.6)	1	1	
Presentation					
Non‐vertex	19 (73.1)	7 (26.9)	7.82 (3.17–19.26)	5.27 (1.83–15.16)	0.002
Vertex	85 (25.8)	245 (74.2)	1	1	

Abbreviations: AOR, adjusted odds ratio; COR, crude odds ratio; PIH, pregnancy‐induced hypertension.

## 4 Discussion

The purpose of this study was to ascertain the magnitude of NNM and related variables in hospitals located in Central Ethiopia's Southwest Shoa Zone. NNM is a relatively new idea that is starting to gain traction as a key metric for monitoring and assessing the standard of prenatal care. Stakeholders and policymakers should assess the scope of NNM and its contributing factors. This provides information to help hospital management and the zonal health department plan and carry out activities meant to improve the perinatal outcome. Additionally, this study will provide evidence that physicians can better understand the extent and contributing factors of the problem and step up their efforts to improve the health of newborns.

It also assists in determining the contributing factors to neonatal morbidity so that appropriate measures can be taken at the community and health system levels.

The magnitude of neonatal near miss was 29.2% (95% CI: 24.47–33.96%), according to the study's findings. The results of other Ethiopian studies, including those at Jimma public hospitals (26.7%), Hawassa governmental hospitals (33.4%), Debretabor general hospital (32.9%), and a Brazilian university (30.33%), are in line with this finding [9, 10, 13, 14]. The observed result, however, is more than the findings of the studies carried out in South Ethiopia (4.5%) and Injibera General Hospital (23.3%) [15, 16]. Furthermore, it is higher than research done in the WHO‐multi‐country study (7.25%), Nepal (7.9%), India (8.8%), and tertiary hospitals in Northeast Brazil (8.6%) [12, 17, 18, 19]. The greater percentage of housewives, rural dwellers, and mothers who did not receive ANC follow‐up in the current study compared to the Injibera study may be the cause of this difference of a greater magnitude of NNM. Among other factors that were taken into consideration, the inclusion and exclusion criteria that were applied may have contributed to the disparities in the Injibera study, which was carried out at a single public hospital. The fact that there were more mothers of newborns in the current study who were housewives and rural dwellers without formal education, as well as variations in the study methodology and inclusion and exclusion criteria, may also be the reason for the discrepancy when compared to the study conducted in South Ethiopia. Another explanation could be that the South Ethiopian study included neonatal mortality, and the newborns were monitored until the seventh postpartum day, sometimes known as the early neonatal phase. The sociodemographic and economic status of the study population (mothers’ health seeking behavior, their ability to recognize complications early, the availability of technologies for early detection and management of complications), as well as the health delivery system and setting, may be the cause of the other inconsistencies in magnitude when compared to some studies from Brazil, Nepal, and India [12, 18, 20, 21, 22, 23].

Additionally, the criteria used to define NNM cases may be the cause of the higher degree of inconsistency. Studies conducted in Brazil and India only used pragmatic criteria to identify NNM cases [20, 22, 23], but the current study used a combination of criteria to identify NNM. The larger discrepancy may be due to differences in inclusion and exclusion criteria, as well as sample size, when compared to WHO's multi‐country survey study [17].

However, compared to earlier research in Ghana (69.5%), Brazil (38.89%), and Uganda (36.7%), the current study indicated a reduced magnitude of NNM [5, 6, 24]. A study in Uganda used neonates of mothers with severe obstetric issues, which may cause the babies to encounter life‐threatening situations and may designate them as NNM instances. This could explain the observed discrepancy of lesser magnitude, which may be caused by different criteria and settings. The pragmatic criteria for NNM identification were used independently in the Brazilian study, which only included neonates admitted to the NICU, and the Ghanaian study, which included neonates with problems and admitted to the NIC.

ANC visits are an independent predictor of NNM, according to the current study. Therefore, compared to neonates born to mothers who received adequate ANC visits, those whose mothers did not receive adequate ANC visits had a 2.6‐fold higher likelihood of experiencing NNM. The research done in Debretabor, India, and Brazil supports this conclusion [13, 25, 26]. This may be explained by the fact that a poor ANC visit results in poor prenatal care (such as advice on identifying and managing diseases and warning signs of pregnancy) and impacts the maternal continuum of care, which in turn impacts the health outcomes of the newborn.

According to the current study, neonates born to mothers with APH had a roughly five‐fold increased odds of developing NNM compared to those delivered to mothers without APH. This result is in line with earlier research, such as that conducted in Brazil and Debretabor [11, 13]. In a similar vein, mothers with PROM were around four times more likely than mothers without PROM to give birth to a child with NNM. Similar studies conducted in Northern, Northwest, and Southern Ethiopia reported similar findings [13, 15, 25]. Some investigations found that PROM was associated with decreased amniotic fluid, which in turn was associated with a non‐reassuring fetal heart rate pattern, asphyxia, premature birth, chorioamnionitis, neonatal sepsis, and indirectly caused intraventricular hemorrhage and Respiratory Distress Syndrome [19, 27].

The odds of NNM were three times higher for mothers with pregnancy‐induced hypertension than for mothers without a history of PIH. This finding is in line with research findings from Jimma, Debretabor, and Gamo Gofa in Ethiopia and Brazil [9, 12, 13, 21, 28]. According to a study done in the Tigray region and the hospitals of the Wolaita Zone in Ethiopia, this could be because of hypertension during pregnancy, which can lead to birth asphyxia and complications like intrauterine growth retardation and preterm birth, which are more likely to have low birth weight [19, 29]. The results of this study, however, contradict those of a study carried out in Hawassa, Southern Ethiopia [10]. Methodological variables could include variations in study area, sampling strategy, and sample size.

The current study found that mothers who gave birth via cesarean section had about three times the odds of acquiring NNM cases as compared to mothers who gave birth naturally vaginally. Other research done in Ethiopia, including in Jimma, Gurage Zone, Gamu Gofa, and Hawassa City, as well as research done in Brazil, supports this finding [9, 10, 22, 30]. This may be because, as studies have shown, cesarean sections have been associated with higher rates of infant morbidity and mortality as well as a delayed or nonexistent improvement in neonatal outcomes when they are not performed for medical reasons [31, 32].

Another explanation is that babies born via cesarean section have less skin‐to‐skin contact with their mothers right after delivery. Because of the anesthesia the mothers received during the procedure, the neonates are unable to start breastfeeding within an hour of birth, increasing the risk of early complications like hypothermia and hypoglycemia [32, 33]. Similarly, an on‐demand elective cesarean section may occasionally increase the chance of preterm birth [34].

Furthermore, compared to neonates with vertex presentation at birth, those with non‐vertex presentation at birth were more likely to develop NNM. This is consistent with research done in southern Ethiopia (Gamu Gofa) and southwest Ethiopia (Jimma) [9, 28]. Complications such as extended labor, delivery hypoxia, cord prolapse, and birth trauma may be the result of malpresentation [35, 36].

### 4.1 Limitations

Since mothers who gave birth outside of hospitals (at health centers or at home) were excluded from the study due to the difficulty of obtaining information about both the mothers (investigations results during ANC visit) and the neonates (5th minute APGAR score, birth weight, gestational age), the fact that the neonates were only sampled from hospitals may have resulted in an underestimation of the magnitude of NNM. The controlled circumstances in this institution‐based study make it difficult to identify complicated social conditions. Institution‐based studies may overlook the many activities and experiences seen in the larger community. This could result in a lack of generalizability and the failure to identify important characteristics that could influence the severity of a newborn near‐miss.

Another limitation is that the follow‐up was limited to the first 24 h after delivery or NICU discharge, rather than the full postnatal period. But throughout the newborn period, certain circumstances could arise at any time and lead to NNM.

## 5 Recommendation

For health planners (Hospital administrators and Zone health office)

- Strengthening adequate antenatal care visits to improve the maternal and neonatal health care services, especially in the early initiation of ANC.
  For health care providers
- Counseling the mothers about obstetric complications is important for early recognition and care seeking.
- Early detection and appropriate and evidence‐based management of obstetric complications.

For researchers

- Further research is needed to identify other risk factors, like evidence‐based care, and timely appropriate management given by using a longitudinal (follow‐up) study design for the full 28 days of postnatal period.

## 6 Conclusion

According to this study, a NNM condition occurred in almost three out of ten neonates born in hospitals in the southwest Shoa Zone of the Oromia area. This magnitude is comparable to Ethiopia's national pooled prevalence of NNM. Neonates born to pregnant women with obstetric problems such as APH, PROM, and PIH with inadequate ANC visits were more likely to experience NNM. Furthermore, neonates with non‐vertex presentation at birth and those delivered via cesarean section had a higher risk of developing NNM.

Health planners and healthcare professionals must work to enhance maternal and neonatal health care services, particularly in the areas of early ANC initiation, early detection, and appropriate, evidence‐based therapy of obstetric problems, to strengthen targeted prenatal care. Furthermore, this study offers evidence to help healthcare professionals comprehend the scope and contributing elements of the issue and to increase their efforts to enhance newborn health. To implement suitable actions at the community and health system level, it also aids in identifying the contributing causes to newborn morbidity.

## Author Contributions

**Shiferaw Benti:** conceptualization; methodology; software; data curation; investigation; formal analysis; supervision; writing – original draft. **Tsegaye Benti Muse:** conceptualization; investigation; writing – original draft; methodology; validation; writing – review and editing; formal analysis; supervision. **Kisu Meskele Telila:** conceptualization; methodology; supervision. **Rahel Abdissa:** investigation; project administration; data curation; resources. **Elias Teferi Bala:** investigation; methodology; formal analysis; supervision; writing – review and editing; writing – original draft.

## Conflicts of Interest

The authors declare no conflicts of interest.

## Data Availability

The data that support the findings of this study are available on request from the corresponding author. The data are not publicly available due to privacy or ethical restrictions.
